# CagA Effector Protein in *Helicobacter pylori*-Infected Human Gastric Epithelium in Vivo: From Bacterial Core and Adhesion/Injection Clusters to Host Cell Proteasome-Rich Cytosol

**DOI:** 10.3390/toxins11110618

**Published:** 2019-10-25

**Authors:** Vittorio Necchi, Vittorio Ricci, Patrizia Sommi, Enrico Solcia

**Affiliations:** 1 Department of Molecular Medicine, University of Pavia, 27100 Pavia, Italy; vittorio.necchi@unipv.it (V.N.); patrizia.sommi@unipv.it (P.S.); solciae@smatteo.pv.it (E.S.); 2 Centro Grandi Strumenti, University of Pavia, 27100 Pavia, Italy; 3 Pathologic Anatomy Unit, Fondazione IRCCS Policlinico San Matteo, 27100 Pavia, Italy

**Keywords:** *Helicobacter pylori*, CagA, human gastric epithelium in vivo, host–pathogen interactions, gastric cancer, ultrastructural immunocytochemistry

## Abstract

A key role in the carcinogenic action of *Helicobacter pylori* is played by the effector protein CagA, the first identified oncoprotein of the bacterial world. However, the present knowledge in regard to the bacterial injection of CagA into epithelial cells (through a type IV secretion system) and its intracellular fate is based primarily on experimental studies in vitro. Our study was aimed to investigate, in *H. pylori*-infected human gastric epithelium, CagA delivery and intracellular distribution in order to identify any in vivo counterpart of the cell injection mechanism described in vitro and any intracellular cytoplasmic site of preferential CagA distribution, thus shedding light on the natural history of CagA in vivo. By transmission electron microscopy and ultrastructural immunocytochemistry (which combine precise molecule localization with detailed analysis of bacterial-host cell interaction and epithelial cell ultrastructure), we investigated endoscopic biopsies of gastric antrum from *H. pylori*-infected dyspeptic patients. Our findings provide support for CagA direct injection into gastric epithelial cells at bacterial adhesion sites located on the lateral plasma membrane and for its cytosolic intracellular distribution with selective concentration inside peculiar proteasome-rich areas, which might be site not only of CagA degradation but also of CagA-promoted crucial events in gastric carcinogenesis.

## 1. Introduction

The Gram-negative bacterium *Helicobacter pylori* infects about half of the world’s population and plays a key role in human carcinogenesis. Classified by the World Health Organization as a class-I carcinogen [1], *H. pylori* infection is the strongest known risk factor for gastric neoplasms [2,3]. Among several different virulence factors produced by the bacterium, a key role in the carcinogenic action of *H. pylori* is played by the effector protein CagA [4,5,6]. Encoded by the *cag* pathogenicity island together with the components of a type IV secretion system (T4SS) devoted to injecting it into target cells, CagA is indeed the first identified bacterial oncoprotein, i.e., a protein playing a well-established role in human carcinogenesis. 

Tegtmeyer et al. [7] recently demonstrated that CagA is delivered to gastric epithelial cells by *H. pylori* penetrating lateral intercellular spaces after disrupting the apical intercellular junctional complex through the serine protease HtrA. Indeed, *H. pylori* interaction with the basolaterally-located integrin-β1 membrane receptor promotes the cellular injection of CagA through the bacterial T4SS [8]. Once inside the gastric epithelial cells, CagA undergoes tyrosine phosphorylation at its Glu-Pro-Ile-Tyr-Ala (EPIYA) motifs by Src and Abl kinases [9] and, according to light microscopy immunofluorescence observations of in vitro cell culture experiments, would concentrate at the inner leaflet of epithelial plasma membrane while acting as a “non-physiological scaffold/hub protein by interacting with multiple host signaling molecules” [5]. At present, no comprehensive investigation has been made on in vivo CagA delivery mechanism or intracellular distribution, including possible interaction with different cell organelles, membranes or cytosolic components, despite its well-known crucial role in human gastric carcinogenesis. Among several disclosed mechanisms of CagA-dependent carcinogenesis, special attention has been paid to CagA direct or indirect interaction with the ubiquitin-proteasome system (UPS) to promote degradation of oncosuppressor gene products like p53, RUNX3 and related factors [10,11,12]. Recently, Abdullah et al. [13] also suggested a role of proteasome, in addition to autophagy, in CagA degradation and showed cytoplasmic accumulation of CagA when proteasome activity was inhibited. Interestingly, we previously identified in vivo and in vitro, in *H. pylori*-infected human gastric epithelium as well as in a variety of neoplastic cells (including gastric cancer), a novel proteasome-rich cytoplasmic structure (PaCS), where CagA also appeared to accumulate [14,15]. 

We thus decided to reinvestigate the precise distribution of CagA in infected human gastric epithelium by ultrastructural immunocytochemistry, looking for: (a) any in vivo counterpart of the cell injection mechanism described in vitro, and (b) any intracellular cytoplasmic site of preferential CagA distribution and CagA–UPS interaction.

## 2. Results

**Figure 1:**
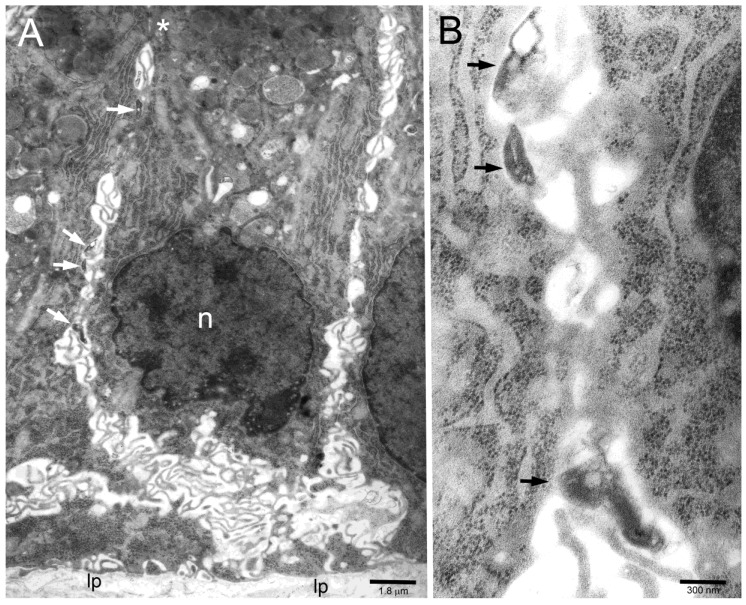
(**A**) Several *H. pylori* (arrows) inside intercellular lateral spaces (note typical undulating membrane plications) of infected human gastric epithelium in vivo. The asterisk marks two subapical desmosomes. n, epithelial cell nucleus; lp, lamina propria. (**B**) Three of the bacteria in (**A**) are enlarged to show their adherence (arrows) to the epithelial cell membrane.

Ultrastructural investigation of gastric antral biopsies from patients known from previous studies to be colonized by *H. pylori* at the level of the gastric luminal surface [16,17] allowed us to detect bacteria infiltrating lateral intercellular spaces of the epithelium, often with patterns of bacterial-to-epithelial cell adhesion (Figure 1A,B). 

**Figure 2:**
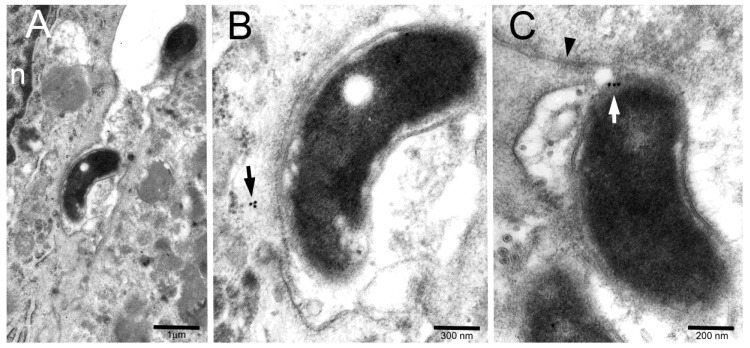
(**A**,**B**) Two intercellular space bacteria (one of which enlarged in (**B**) to improve identification of immunogold particles) show CagA in their core. A small cluster of CagA immunoreactivity (arrow in (**B**)) is also visible in the submembranous cytoplasm of a bacterium-adhering cell. n, epithelial cell nucleus. (**C**) A bacterium, lying just below a tight junction (arrowhead), shows a CagA immunogold cluster (white arrow) across its periplasm and epithelial adherence site.

The immunogold technique showed CagA reactivity in the majority of tested bacteria, either in the core or more peripherally, at the site of cell adhesion (Figure 2A–C). 

**Figure 3:**
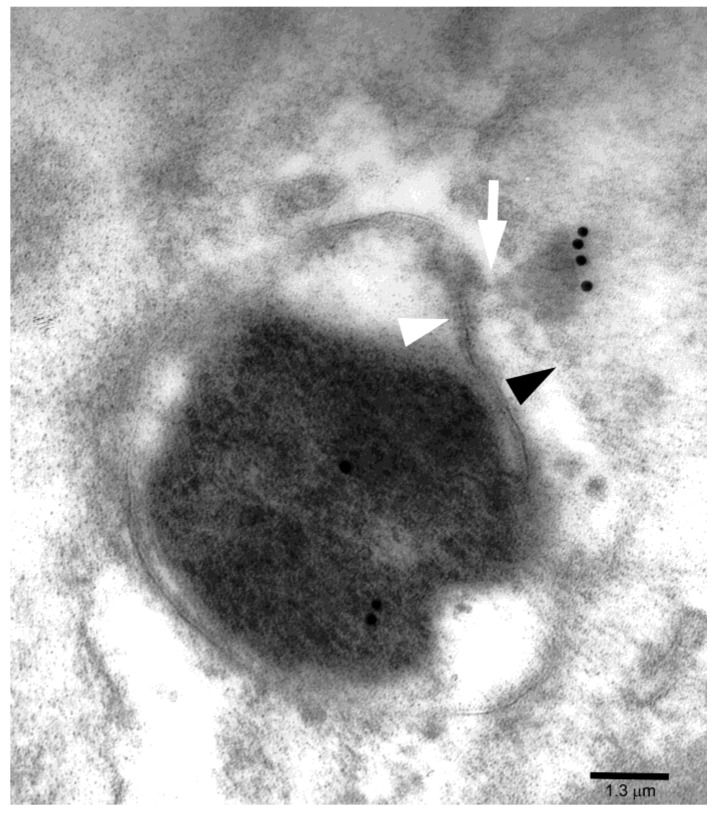
Another intercellular bacterium shows CagA immunogold in its core as well as on the cytoplasmic front of a relatively dense focal structure crossing the epithelial membrane (black arrowhead) while retaining structural connection (white arrow) with bacterial outer membrane (white arrowhead).

Occasionally, minute CagA clusters were also detected in the underlying submembranous cytoplasm of adherent epithelial cells (Figure 2B) or even on the cytosolic front of fairly dense material entering the cell while still retaining physical connection with the bacterial outer membrane (Figure 3).

**Figure 4:**
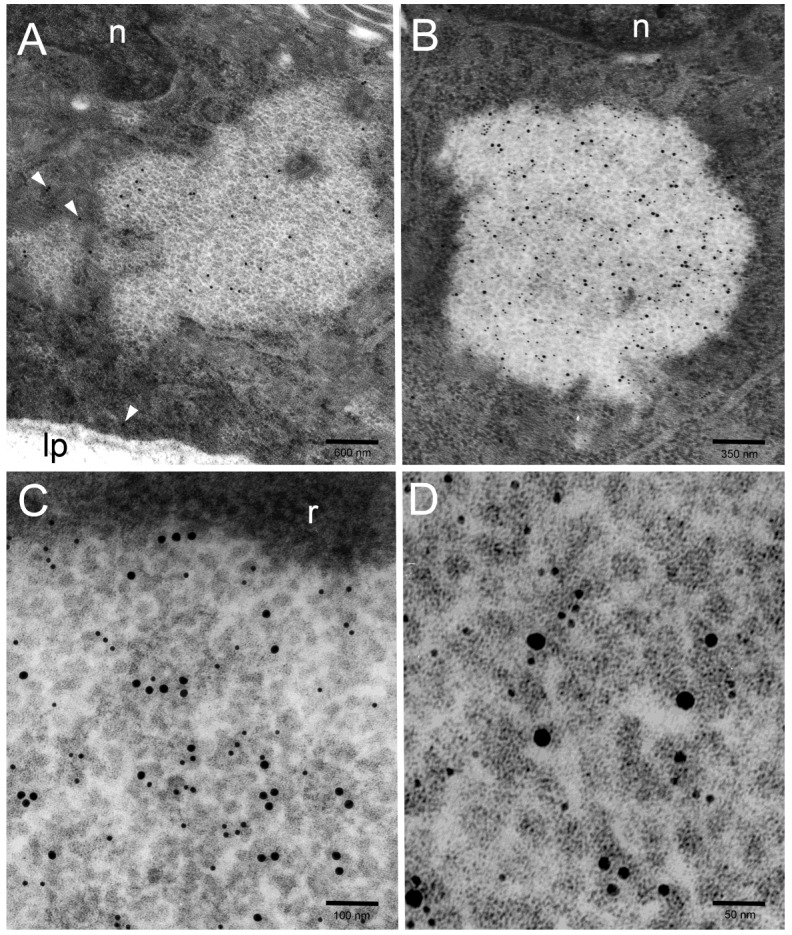
(**A**) CagA immunoreactivity in the basal cytoplasm of an epithelial cell showing intense immunogold deposition over clear, irregularly shaped proteasome particle-rich cytoplasmic structure (PaCS) areas. Note CagA reactivity of minute PaCSs arising focally in the cytosol interposed between PaCS-surrounding ribosomes. Also note sparse minute CagA deposits (arrowheads) in the cytosol adjacent to the rough endoplasmic reticulum or free ribosomes. n, epithelial cell nucleus; lp, lamina propria. (**B**,**C**) Double CagA (smaller gold particles) and 19S proteasome (larger gold particles) immunolabelling identifies the clear, cytoskeleton-poor PaCS as a CagA- as well as proteasome-rich structure. Note that PaCS-surrounding ribosomes in (**B**) and on top of (**C**) differ in electron density and shape from the proteasome-reactive barrel-like particles filling the PaCSs. r, ribosomes. (**D**) The barrel-like shape and thinly punctate high-resolution pattern of 20S proteasome-reactive particles (5-nm gold particles) is recognized when under favorable cutting plan. Sparse CagA (15-nm gold particles) and polyubiquitinated proteins (10-nm gold particles) immunoreactivities are also visible.

A prominent CagA immunoreactivity (Figure 4) was often found in areas of basal (i.e., below the nucleus) cell cytoplasm characterized by a collection of barrel-like particles, which showed proteasome immunoreactivity when tested with double CagA/proteasome immunogold tests (Figure 4B–D), thus characterizing such areas as proteasome particle-rich cytoplasmic structures or PaCSs. Notably, CagA immunoreactivity was also seen in minute PaCSs forming in the cytosol as sparse deposits interposed in between ribosomal particles (Figure 4A). PaCS particles differed from surrounding ribosomes in shape, structure and density. In addition, well-formed PaCS areas substantially lacked cytoplasmic organelles like mitochondria, endosomes, lysosomes or endoplasmic reticulum, as well as most cytoskeleton components.

**Figure 5:**
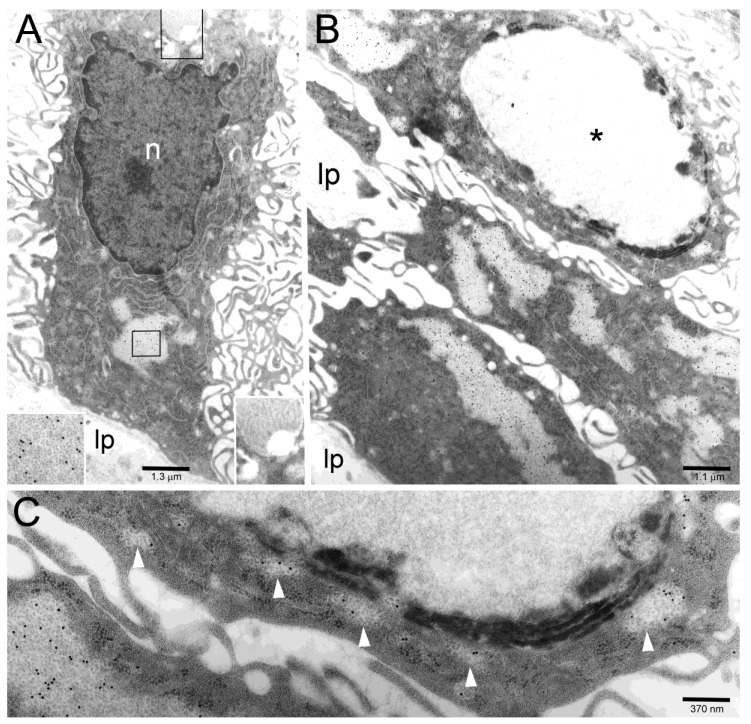
(**A**) A columnar epithelial cell shows CagA-reactive PaCSs in its basal part (enlarged in the left lower corner inset to help recognizing gold particles inside PaCSs) and CagA-unreactive vacuolar/vesicular structures in its supranuclear part (enlarged in the right lower corner inset to better recognize the absence of immunogold particles). n, epithelial cell nucleus; lp, lamina propria. (**B**,**C**) Three cells in a tangentially cut epithelium show several CagA-positive PaCSs, to be compared with a single CagA-negative large autophagolysosomal body (asterisk) in the upper cell, which is enlarged in (**C**) to improve identification of the immunogold particles inside PaCSs (including minute PaCSs, see arrowheads, sparse in the cytoplasm surrounding the autophagolysosomal body) as well as of dense, osmiophilic deposits and membranes in the autophagolysosomal body.

As a rule, no consistent CagA immunoreactivity was detected inside membrane-limited vacuoles, autophagic vesicles or the autophagolysosomal bodies (Figure 5) we frequently found in *H. pylori*-infected gastric epithelial cells, especially in their supranuclear cytoplasm, and characterized by VacA, p62/SQSTM1, LC3 and cathepsin D immunoreactivity, as described in a previous paper [17].

## 3. Discussion

This in vivo study provides ultrastructural immunocytochemistry evidence for CagA delivery, by *H. pylori* penetrated into gastric epithelium lateral intercellular spaces, to nearby adhering epithelial cells. Inside the bacterium, CagA appeared mainly localized into its core cytoplasm; however, focal clusters were seen at periplasmic/membrane level at sites of bacterial-to-epithelial cell adhesion. We obtained images suggestive for a discrete delivery of CagA immunoreactive material to the interacting cell, up to its immediately submembranous cytoplasm. These pictures may fit with the T4SS injection mechanism identified in co-culture experiments in vitro and by scanning electron microscopy [7,18,19]. In addition, our analysis of chronically-infected human gastric epithelium shows that CagA immunoreactivity may also appear in the cytoplasm away from the cell membrane, either as sparse minute deposits in the cytosol adjacent to the rough endoplasmic reticulum and free ribosomes or, more prominently, inside particular cytoplasmic areas characterized by a collection of proteasome-reactive particles. These areas represent the so-called PaCSs described in *H. pylori*-infected human gastric epithelium in previous papers [14,15,20]. It must be outlined that PaCSs are poorly preserved by the short formaldehyde fixation commonly used in immunofluorescence experiments, whereas being optimally preserved by the combined aldehyde-osmium fixation procedure used in our ultrastructural, and parallel light microscopy, immunocytochemical investigations [20,21]. 

PaCSs, which so far have been observed in various types of neoplastic and embryonic-fetal cells, though not in normal uninfected adult cells [22,23], are also known to store polyubiquitinated proteins and heat-shock proteins 70 and 90 [24,25]. Thus, they seem ideally equipped to be a potential interaction site between CagA and UPS, an interaction known to be crucial for CagA-promoted gastric carcinogenesis [10,11,12]. Confirming previous findings [14,15], our localization of CagA inside an UPS-rich cytosolic area like PaCS may help clarify the mechanism of proteasome-dependent, CagA-promoted degradation of oncosuppressor proteins.

The similarity of CagA action on cultured gastric epithelial cells (including cell scattering and elongation) with that obtained on the same cells using trophic factors such as the hepatocyte growth factor (HGF) led to the hypothesis that CagA may aberrantly activate some intracellular signaling usually triggered by growth factors activating ERK1,2/MAPK kinases and related proliferative responses [5,26,27]. This hypothesis seems of interest as some of such factors have been shown to play a role in the development of PaCSs in vitro (cytokines like GM-CSF and several interleukins) and in vivo (fibroblast growth factor pathway) [15,20,21,24], in addition to promoting carcinogenesis [3,15,28]. Indeed, the interaction of CagA with trophic factors, confirmed by its rapid intracellular association with, and activation of, c-Met (i.e., the receptor of HGF) [29,30,31] and by its capacity to transactivate the EGF receptor with resulting ERK1,2 activation [28,32], may link together two of the main pathways involved in gastric carcinogenesis, i.e., UPS-dependent oncosuppressor degradation and trophic factor activation.

The intracellular fate of CagA still remains an open question. Tsugawa et al. [33] reported that, in vitro, CagA was degraded by autophagy, which may be specifically triggered by VacA toxin. More recently, in vitro results by Abdullah et al. [13] suggested that both autophagy and proteasome (UPS) have a role in CagA degradation and that VacA toxin would cause CagA sequestration and accumulation in dysfunctional autophagosomes because of the disrupting action of the toxin on late steps of the autophagic pathway. In our in vivo study presented here, we found no consistent evidence of CagA immunoreactivity inside autophagolysosomal bodies and related vacuolar-to-dense structures entering the bodies. The autophagolysosomal bodies were frequently detected in gastric epithelium chronically infected by *H. pylori* and, in previous investigations, showed VacA toxin immunoreactivity in addition to autophagic-lysosomal markers like p62/SQSTM1, LC3 or cathepsin D [17]. In general, in our gastric biopsies from *H. pylori*-infected patients we found a limited evidence of VacA and CagA colocalization, among which a moderate/focal immunoreactivity for VacA inside PaCSs, especially when the latter were merging with VacA-carrying endoplasmic reticulum cisternae [17]. While VacA was previously shown mainly to concentrate inside membrane-limited compartments such as endocytic vesicles, endosomes, and related VacA-induced vacuoles or autophagolysosomes [17], CagA was shown here to remain essentially cytosolic (including PaCS, an organelle-poor cytosolic compartment not limited by membranes). However, it should be outlined that it may be difficult to reconstruct in chronic human pathologic conditions the structural and functional counterpart of relatively short-term experiments in vitro, especially concerning reciprocal VacA/CagA interactions possibly mediated by “third” molecules, also considering the relatively short intracellular half-life (about 200 min) of CagA molecule [34]. 

In conclusion, in this investigation we tried to reconstruct the natural history of CagA effector protein in vivo. Our ultrastructural cytochemical data provide support for CagA direct injection into gastric epithelial cells at bacterial adhesion sites located on the lateral plasma membrane. The cytosolic intracellular distribution of CagA showed selective concentration inside proteasome-rich areas, which might be site not only of CagA degradation but also of CagA-promoted crucial events in gastric carcinogenesis.

## 4. Materials and Methods 

### 4.1. Human Biopsy Samples

We reinvestigated by transmission electron microscopy (TEM) the endoscopic biopsies of gastric antrum from 15 dyspeptic patients, which, in previous investigations [17], were found to be extensively *H. pylori*-infected at the level of their luminal surface and in the absence of neoplasia or dysplasia. Gastric antral biopsies from subjects showing *H. pylori*-negative, non-neoplastic, non-metaplastic, uninflamed mucosa were taken as controls. The study was conducted in accordance with the Declaration of Helsinki and the protocol was approved by the Ethics Committee of Fondazione IRCCS Policlinico San Matteo (Pavia, Italy; project identification code: P-20020001513) as a reinvestigation of archival material along the same line (i.e., diagnosis of *H. pylori*-dependent gastritis) as for the original written consensus.

### 4.2. TEM and Ultrastructural Immunocytochemistry 

For TEM investigation, biopsy samples were fixed for 4 hours with 2% formaldehyde and 2.5% glutaraldehyde in 0.1 M phosphate buffer (pH 7.3), followed by 1% osmium tetroxide for 1 hour, and then embedded in Epon-Araldite resin [14,17]. Thin (~70 nm) sections were stained with uranyl-lead or underwent the immunogold procedure followed by uranyl-lead counterstaining, as described previously [16,17,20]. Double immunogold procedures were performed by using: (a) two primary Abs obtained from different animal species (i.e., rabbit or mouse), followed by pertinent secondary Abs marked by gold particles of different size, or (b) two primary Abs raised in the same animal species and applied separately to the two faces of the same thin resin section (collected on a 300-mesh grid), followed by appropriately marked secondary Ab. Triple immunogold labelling was obtained by combining these two procedures. Specimens were analyzed by a Jeol JEM-1200 EX II transmission electron microscope equipped with an Olympus CCD camera (Mega View III). Images were processed and assembled by using the Adobe Photoshop CS5 software. 

### 4.3. Antibodies

CagA immunogold reactivity of bacteria and epithelial cells was tested on resin sections by using the following primary Abs: (1) rabbit polyclonal HPP-5003-9 or mouse monoclonal HPM-5001-5 Abs (Austral Biologicals, San Ramon, CA, USA) raised against a highly purified recombinant CagA antigen; (2) rabbit polyclonal sc-25766 Ab (Santa Cruz Biotechnology, Santa Cruz, CA, USA) raised against a recombinant CagA fragment (aminoacids 1–300); (3) rabbit polyclonal Ab raised against recombinant CagA [35], kindly given by Dr. A. Covacci (Siena, Italy). In addition, possible CagA colocalization with proteasome (both 19S and 20S molecular species) and polyubiquitinated proteins, two well-established markers of PaCSs [20,22], were tested by using: (1) rabbit polyclonal 539166 Ab (Calbiochem, La Jolla, CA, USA) raised against 19S proteasome S2 subunit; (2) rabbit polyclonal BML-PW8155 Ab (Enzo Life Sciences, Farmingdale, NY, USA) raised against 20S proteasome α/β subunits; (3) mouse monoclonal FK1 Ab (BML-PW8805; Enzo Life Sciences) raised against polyubiquitinated proteins. As secondary Abs, anti-rabbit or anti-mouse immunoglobulins labelled with 5 to 20 nm colloidal gold particles (British Bio Cell, Cardiff, UK, and Aurion, Wageningen, The Netherlands) were used [17].

Tests to evaluate the specificity of immunogold labelling were carried out using antibodies absorbed with excess antigen and omitting or substituting the specific antibodies in the first layer of the immunogold procedure. Positive and negative controls were obtained by parallel investigation of *H. pylori* cultures, epithelial cell cultures, and *H. pylori*-positive or -negative gastric mucosa specimens as in previous studies [16,17,20]. In particular, anti-CagA Abs used were tested by parallel TEM investigation on well-characterized bacterial cultures either CagA-producing (*H. pylori* strains 60190, ATCC 49503, and CCUG 17874, from Culture Collection University of Göteborg, Sweden) or not producing CagA *(H. pylori* strain 60190:M22, the isogenic mutant of the 60190 strain in which the *cagA* gene was disrupted by insertional mutagenesis; kindly provided by Dr. T.L. Cover, Nashville, TN, USA), fixed and embedded as done for *H. pylori*-colonized biopsies. We found that the rabbit polyclonal HPP-5003-9 Ab from Austral Biologicals gave the best results in term of sensitivity, specificity and reproducibility. All the pictures here shown were obtained with such an Ab.
